# Parietal epithelial cell differentiation to a podocyte fate in the aged mouse kidney

**DOI:** 10.18632/aging.103788

**Published:** 2020-08-28

**Authors:** Natalya V. Kaverina, Diana G. Eng, Jeffrey H. Miner, Jeffrey W. Pippin, Stuart J. Shankland

**Affiliations:** 1 Division of Nephrology, University of Washington, Seattle, WA 98195, USA; 2 Division of Nephrology, Washington University School of Medicine, St Louis, MO 63110, USA

**Keywords:** reporter, tdTomato, EGFP, differentiation, Bowman's capsule

## Abstract

Healthy aging is typified by a progressive and absolute loss of podocytes over the lifespan of animals and humans. To test the hypothesis that a subset of glomerular parietal epithelial cell (PEC) progenitors transition to a podocyte fate with aging, dual reporter *PEC-rtTA|LC1|tdTomato*|*Nphs1-FLPo*|*FRT-EGFP* mice were generated. PECs were inducibly labeled with a tdTomato reporter, and podocytes were constitutively labeled with an EGFP reporter. With advancing age (14 and 24 months) glomeruli in the juxta-medullary cortex (JMC) were more severely injured than those in the outer cortex (OC). In aged mice (24m), injured glomeruli with lower podocyte number (41% decrease), showed more PEC migration and differentiation to a podocyte fate than mildly injured or healthy glomeruli. PECs differentiated to a podocyte fate had ultrastructural features of podocytes and co-expressed the podocyte markers podocin, nephrin, p57 and VEGF164, but not markers of mesangial (Perlecan) or endothelial (ERG) cells. PECs differentiated to a podocyte fate did not express CD44, a marker of PEC activation. Taken together, we demonstrate that a subpopulation of PECs differentiate to a podocyte fate predominantly in injured glomeruli in mice of advanced age.

## INTRODUCTION

Glomerular podocytes are terminally differentiated epithelial cells that cannot proliferate, and are therefore unable to self-renew [1, 2] Thus, in states of loss such as healthy aging [3–5] and glomerular diseases [6–8] any podocyte replacement must rely on local progenitor/stem cells [9]. Despite evidence that podocytes can be partially or even fully replaced focally, in individual glomeruli in which they have been depleted [10–15], some dispute this notion [16–18]. These differing views might in part be due to the interpretation of results, a focus on density rather than absolute number of new podocytes, the belief that kidney function must be completely restored to prove that even partial replacement in individual glomeruli occurs, and changes in cell type markers that are not always reliable.

Because of the challenges of studying podocyte replacement in humans, studies have largely relied on lessons learned from animal models. This too has contributed to the “podocyte regeneration/replacement debate”. For instance, studies characterized by glomerular hypertrophy that leads to reduced podocyte density without a loss of podocytes themselves should be questioned as a proper model to study podocyte regeneration. Similarly, factors such as mouse age, strain, sex, and the duration of the studies, are potential confounders in this debate. Additionally, the norm is to examine outer cortical glomeruli, but in many instances the juxta-medullary glomeruli are not examined, yet have more pronounced injury [19]. Healthy aging is typified by a progressive and absolute loss of podocytes over the lifespan of animals [18, 20] and humans [21]. Podocyte density is also lowered with advancing age due to both a decrease in absolute number, and a concomitant increase in the size of the glomerulus secondary to aging associated hypertrophy [22]. The age of a mouse is critical in the definition of aging (https://www.nia.nih.gov/research/dab/aged-rodent-colonies-handbook/strain-survival-information). For example, mouse ages 12, 18, and 24 months correspond to human ages 42.5, 56 and 69 years old respectively [23] and therefore using mice aged 17 months and younger is not considered a model of aging.

Currently, glomerular parietal epithelial cells (PECs) [10–12, 24] and cells of renin lineage [13, 25–28] have been shown to acquire many podocyte-like features in experimental models of podocyte depletion. However, we have shown that both sources of podocyte progenitors are adversely impacted by advancing age. For example, their numbers decrease with age leading to a reduced reservoir available to perform progenitor functions [19, 29], they lose some of their characteristic markers, acquire phenotypic changes [19, 30], and their expected responses to stimuli are reduced, such as the response of cells of renin lineage to RAAS blockade [31].

Damage to glomeruli is focally distributed in the aged kidney (defined as less than 50% of all glomeruli), rather being a diffuse phenomenon. Moreover, changes differ in glomeruli of the kidney outer cortex versus the glomeruli of the juxta-medullary region [19]. In order to better understand podocyte replacement within individual glomeruli in healthy middle and advanced aged mice in the context of PEC progenitors, we studied a dual PEC – Podocyte (PEC-PODO) reporter mouse [24] to genetically lineage trace any podocyte replacement from a PEC progenitor source.

## RESULTS

### Glomerular injury is limited to those outer cortical (OC) and juxta-medullary cortical (JMC) glomeruli with reduced podocyte number in aged dual reporter mice

**Figure 1:**
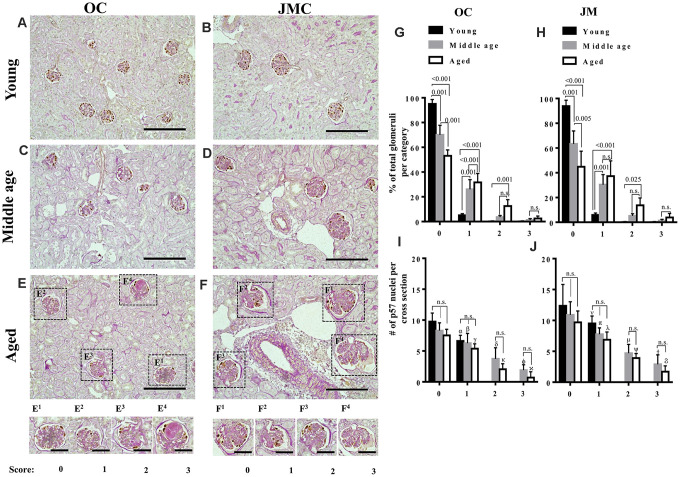
**Glomerulosclerosis in aged PEC-PODO reporter mice.** (**A**–**F**) Representative images of p57 stained (brown, nuclear) podocytes with periodic acid–Schiff staining (PAS) counterstaining from young (6 months) (**A**, **B**), middle age (14 months) (**C**, **D**) and aged (24 months) (**E**, **F**) mice. Glomeruli were divided into two compartments: outer cortex (OC) (**A**, **C**, **E**) and juxta-medullary cortex (JMC) (**B**, **D**, **F**). Small insets represent examples of OC glomeruli (**E^1^**–**E^4^**) and JMC glomeruli (**F^1^**–**F^4^**) for each scoring category that characterized the severity of injury: starting from 0 (no injury) to 3 (globally sclerotic glomeruli). (**G**) Graph of glomerular injury scores in OC. The highest percentage of uninjured glomeruli (score 0) was in young mice (black bars). Middle age mice (gray bars) showed a significantly higher percentage of injured glomeruli (score- 1, 2) and increase in the severity of injury (score-3). Aged mice (white bars) showed a significantly higher percentage of injured glomeruli (score-1,2), and an increase in the severity of injury (score-3) compared to young and middle age mice, while the percentage of uninjured glomeruli was significantly decreased in aged versus young and middle age mice. (**H**) Graph of glomerular injury scores in the JMC. The percentage of injured glomeruli (score-1, 2) was significantly higher in aged mice (white bars) compared to middle age (gray bars) and young (black bars) mice. The percentage of severely injured glomeruli (score-3) was increased but did not reach statistical significance. Middle age mice showed a significant increase in injured (score-1,2) and severely injured glomeruli (score-3) compared young mice. (**I**) Quantification of podocyte number in the OC. Podocyte number per cross section, identified by p57^+^ cells, showed a decreasing trend for middle age and aged mice compared to young mice for individual injury scores, but the differences were not statistically significant. Podocyte number was significantly lower for injured glomeruli (score-1,2,3) compared to uninjured glomeruli (score-0). (**J**) Quantification of podocyte number in the JMC. Podocyte number per cross section, identified by p57^+^cells was higher compared to OC and showed a trend decreasing trend for middle age and aged mice compared to young mice for individual injury scores, but the differences were not statistically significant. Podocyte number was significantly lower for injured glomeruli (score-1,2,3) compared to uninjured glomeruli (score-0).

Because changes in glomeruli in the aging mouse kidney are focal (less than 50% of glomeruli affected), we began by quantitating the degree of damage to individual OC and JMC glomeruli, graded on a score of 0-3 (Figure 1). Silver staining was used to validate the PAS (Supplementary Figure 1). To correlate glomerular injury with podocyte number in individual glomeruli, the number of p57 stained cells per glomerular cross section was also quantitated in each individual glomerulus in which the injury score was recorded (Figure 1). As expected, the overwhelming majority of OC and JMC glomeruli in young mice (6 months) had a injury score of zero, 95.0±3.7% and 93.8±4.7% in OC and JMC respectively (Figure 1A, 1B, 1G, 1H). Interestingly, podocyte number was lower in the occasional glomerulus with a score of 1 in both OC and JMC glomeruli in young mice.

In middle aged mice (14 months), 70.0±7.8% OC and 63.3±10.5% JMC glomeruli had injury scores of zero (Figure 1G, 1H). The remaining glomeruli had scores of 1 (26.0±7.8%), 2 (3.6±1.2%), and 3 (1.2±1.2%) in OC glomeruli (Figure 1C, 1G), and scores of 1 (30.4±8.2), 2 (5.9±1.6%), and 3 (1.3±1.0%) in JMC glomeruli (Figure 1D, 1H). Podocyte number per glomerular cross section was progressively lower with increasing injury scores in both OC and JMC glomeruli (Figures 1I, 1J).

In aged mice (24 months), the percentage of normal glomeruli (score=0) decreased to 53.1±4.7% and 44.9±12.6% in the OC and JMC respectively (Figures 1E–1H). The remaining aged OC glomeruli had scores of 1 (31.6±7.2%), 2 (12.5±5.2%) and 3 (2.4±2%), and the remaining aged JMC glomeruli had scores of 1 (37.3±12.4%), 2 (13.7±5.9%) and 3 (3.9±3.2%). Podocyte number per glomerular cross section was progressively lower in both aged OC and JMC glomeruli with each increase in injury score (Figures 1I, 1G).

### Genetic confirmation that a small fraction of labeled PECs differentiate to a podocyte fate in glomeruli with reduced podocyte number

**Figure 2:**
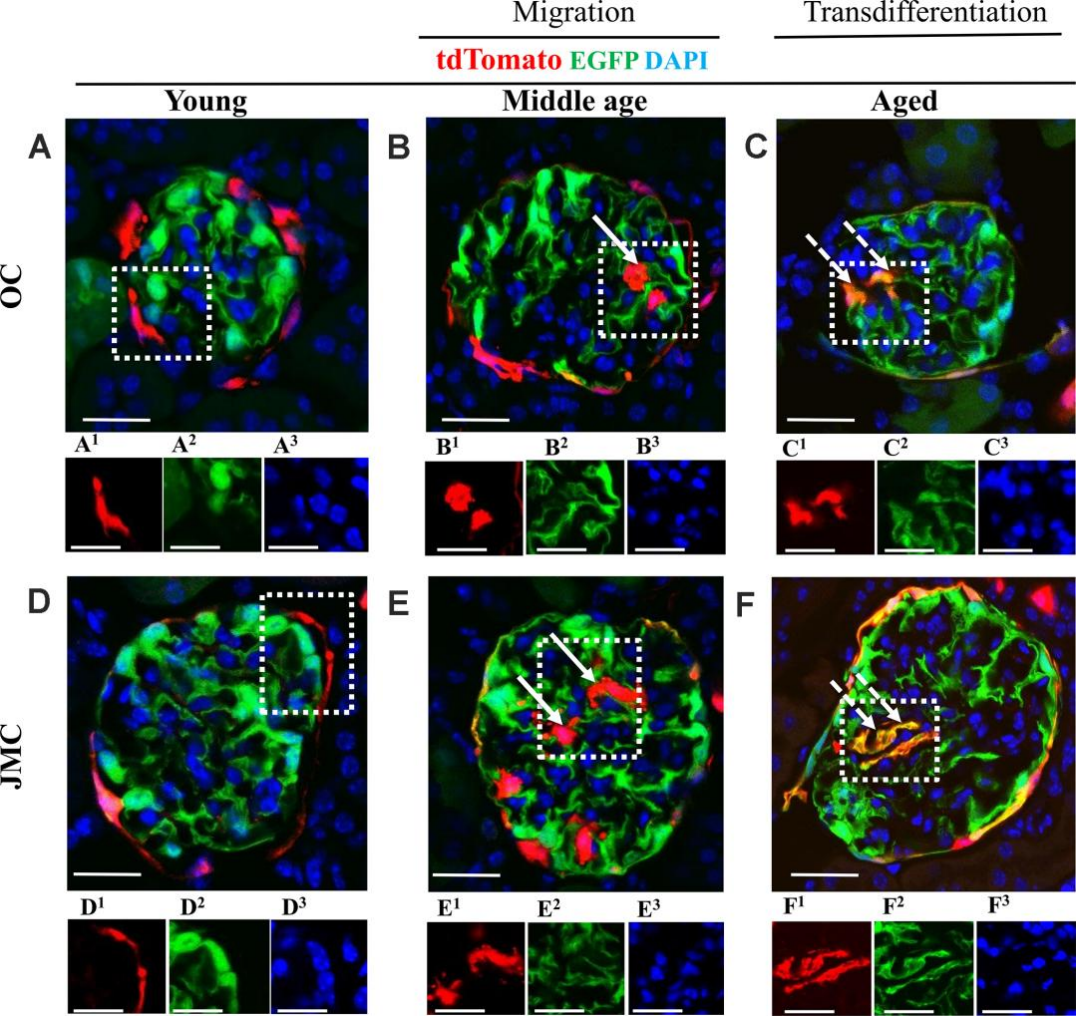
**Migration and differentiation of tdTomato^+^PECs to a podocyte phenotype in aged kidneys.** (**A**–**F**) Representative confocal images of tdTomato-labeled PECs (red), EGFP^+^ labeled podocytes (green), and DAPI stained nuclei (blue) in young, middle age and aged mice in OC (**A**–**C**) and JMC (**D**–**F**). Individual red (labeled with superscript-1), green (superscript-2) and far-red (superscript-3) fluorescent channels of the glomerulus, outlined by the white dashed box. (**A**) Young mice (OC) showed that tdTomato^+^PECs are detected along Bowman’s capsule (**A^1^**) and EGFP^+^ cells (**A^2^**) localized in typical podocyte distribution. Nuclei were labeled with DAPI (**A^3^**). (**B**) Middle age mice (OC) showed that tdTomato^+^PECs (marked with solid arrow) were detected in the glomerular tuft (**B^1^**) with accompanied decrease in the EGFP signal (**B^2^**) and the nuclear marker DAPI (**B^3^**). (**C**) Aged mice (OC) showed that tdTomato^+^PECs (marked with dashed arrow) (**C^1^**) differentiated to a podocyte fate and co-expresses EGFP (green) (**C^2^**), overlap creates a yellow. (**D**) Young mice (JMC) showed that tdTomato^+^PECs are detected along Bowman’s capsule (**D^1^**) and EGFP^+^ podocytes are observed in the glomerular tuft (**D^2^**). (**E**) Middle age mice (JMC) showed that tdTomato^+^PECs (marked with solid arrow) are observed in the glomerular tuft (**E^1^**) with no overlap with EGFP (**E^2^**). (**F**) Aged mice (JMC) showed that tdTomato^+^PECs (marked with dashed arrow) (**F^1^**) overlap with EGFP (**F^2^**) and create a yellow color in the glomerular tuft. Scale bars represent 25μm or 5μm (insets).

The transgenic mice used were designed to visualize permanently undifferentiated PECs (red), native podocytes (green) and new podocytes that derived from a PEC fate (yellow). Autofluorescence of these reporters avoided the need for antibodies for visualization (Figure 2). Although absent in young mice (Figure 2A, 2D), a subset of red PECs were visualized in the glomerular tuft in middle-aged and aged mice (Figure 2B, 2C, 2E, 2F), consistent with migration from Bowman’s capsule. Yellow cells were also detected in the glomerular tuft predominantly in aged mice (Figure 2C, 2F), consistent with differentiation to a podocyte fate.

In order to quantitate the number of undifferentiated labeled PECs (red color) and PECs that differentiated to a podocyte fate (yellow color) in the glomerular tuft, and correlate this with the number of podocytes in the same individual glomerular cross sections in the OC, and in the JMC, staining for p57 and PAS were performed on the same slides in which red, green and yellow fluorescence were observed (Supplementary Figure 2).

**Figure 3:**
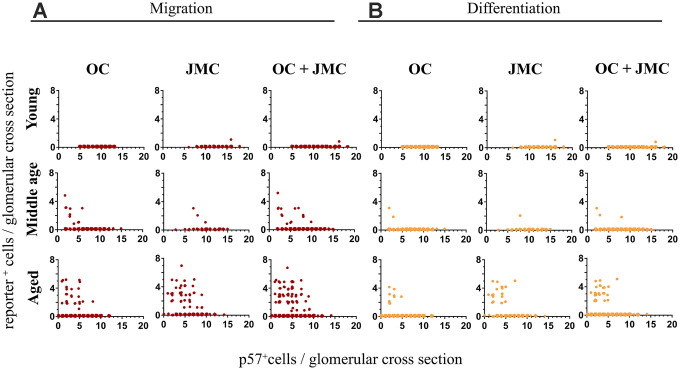
**Quantitative analysis of migration and differentiation of tdTomato^+^PECs to a podocyte fate in aged kidneys.** (**A**) Graphs showing the migration of tdTomato⁺PECs (red cells, y-axis) plotted against the number of podocytes per glomerulus (p57^+^ cells, x-axis) in young, middle age and aged mice in the OC, JMC and both compartments combined (OC and JMC). *Young mice*: There was no migration of tdTomato⁺PECs in the OC, and only one event of migration of a tdTomato^+^PEC to the glomerular tuft in JMC. When OC and JMC were combined, there was only the one event of migration accordingly. *Middle age mice*: The number of glomeruli with migration of tdTomato^+^PECs increased compared to young mice. There was a higher number of glomeruli with migration in the OC compared to JMC. The total increase in migration was reflected when OC and JMC were combined. The glomeruli with migration events tended to be higher in glomeruli with lower podocyte numbers. *Aged mice*: The number of glomeruli with migration of tdTomato^+^PECs to the glomerular tuft was the highest in aged mice compared to young and middle age mice. The incidence of migration predominantly increased in JMC versus OC, and the total increase in migration was reflected when OC and JMC were combined. Again, glomeruli with migration events tended to be higher in glomeruli with lower podocyte numbers. (**B**) Graphs showing the differentiation of tdTomato^+^PECs (yellow cells, y axis) plotted against the number of podocytes per glomerulus (p57^+^ cells, x-axis) in young, middle age and aged mice in the OC, JMC and both compartments combined (OC and JMC). *Young mice*: There was no differentiation of tdTomato⁺PECs (tdTomato^+^EGFP^+^) observed in the OC, and only one event of differentiation in one glomerulus in JMC glomeruli. When OC and JMC were combined, there was only the one event of differentiation accordingly. *Middle age mice*: The number of glomeruli with differentiation of tdTomato⁺PECs (tdTomato^+^EGFP^+^) increased compared to young mice. There were slightly more glomeruli with differentiation in the OC compared to JMC. The total increase in differentiation was relatively low and was reflected when OC and JMC were combined. The glomeruli with differentiation events tended to be higher in glomeruli with lower podocyte numbers. *Aged mice*: The number of glomeruli with differentiation of tdTomato⁺PECs (tdTomato^+^EGFP^+^) was the highest in aged mice compared to young and middle age mice. The incidence of differentiation increased in both OC and JMC glomeruli, and the total increase was reflected when OC and JMC were combined. Again, glomeruli with differentiation events tended to be higher in glomeruli with lower podocyte numbers.

For each individual glomerulus, the number of TdTomato^+^ (red) or TdTomato^+^/EGFP^+^ (yellow) cells per glomerular cross section were plotted against the p57^+^ cell count (used as a podocyte marker) in that same glomerulus. The trend that emerges shows the prerequisite of podocyte loss, and significant podocyte loss, in order for migration and subsequent differentiation to occur (Figure 3). In this particular case, when the number of p57^+^ cells per glomerular cross section drops below 10, red PECs begin to migrate onto the glomerular tuft. In individual glomeruli in which the number of p57^+^ cells drop to below 7, a subset of PECs differentiate to a podocyte-like fate (de novo express EGFP and become yellow). In the glomeruli with podocyte loss that was great enough to warrant migration (red cells on the tuft), 42.0% of those glomeruli experienced differentiation to a podocyte-like fate as defined by the presence of a yellow cell.

Examining the differences between OC and JMC glomeruli independently, in young 6 month-old mice, there was only one glomerulus with migration and differentiation (yellow cell), occurring in a glomerulus in the JMC. This represented 0.1% of all glomeruli, and 0.6% of all JMC glomeruli quantified. No migration or differentiation were noted in the OC glomeruli in young mice. In middle-aged mice, there was a higher percentage of glomeruli with migration (2.6%) and differentiation (0.6%), which occurred in a greater percentage of JMC vs. OC glomeruli. Within OC glomeruli, 2.1% had at least one migration event and 0.4% had at least one differentiation event. JMC glomeruli had a higher incidence of both migration (6.7%) and differentiation (1.7%). At middle-age, 1.4% of glomeruli with at least one migration event also had a differentiation event (20.0% in OC and 25.0% in JMC). In aged mice, the incidence of migration (4.0%) and differentiation (1.7%) increased over middle-age for all glomeruli. In the OC, 1.9% of glomeruli showed at least one migration event, while 0.7% of glomeruli showed at least one differentiation event. In the JMC, 17.9% showed at least one migration event, with 7.7% having at least one differentiation event, indicating the percentage of glomeruli with differentiation occurring on a yes/no basis was 42.2%. In the OC, 38.5% of glomeruli with a case of migration, also experienced differentiation, compared with 44.7% in JMC glomeruli.

Taken together, this data shows that with aging, there is an increase in both the migration and differentiation events on a per glomerulus basis. The total percentage of involved glomeruli increases, but this increase is disproportionally split between the OC and JMC glomeruli, with JMC glomeruli having a much higher percentage of involvement. However, in terms of differentiation, despite the total percent of differentiation on a per glomeruli basis increasing with age, the JMC glomeruli only have a slight increase in the percentage considered as a whole to have had a differentiation event.

### Aged mice have higher extracellular matrix accumulation along Bowman’s capsule

**Figure 4:**
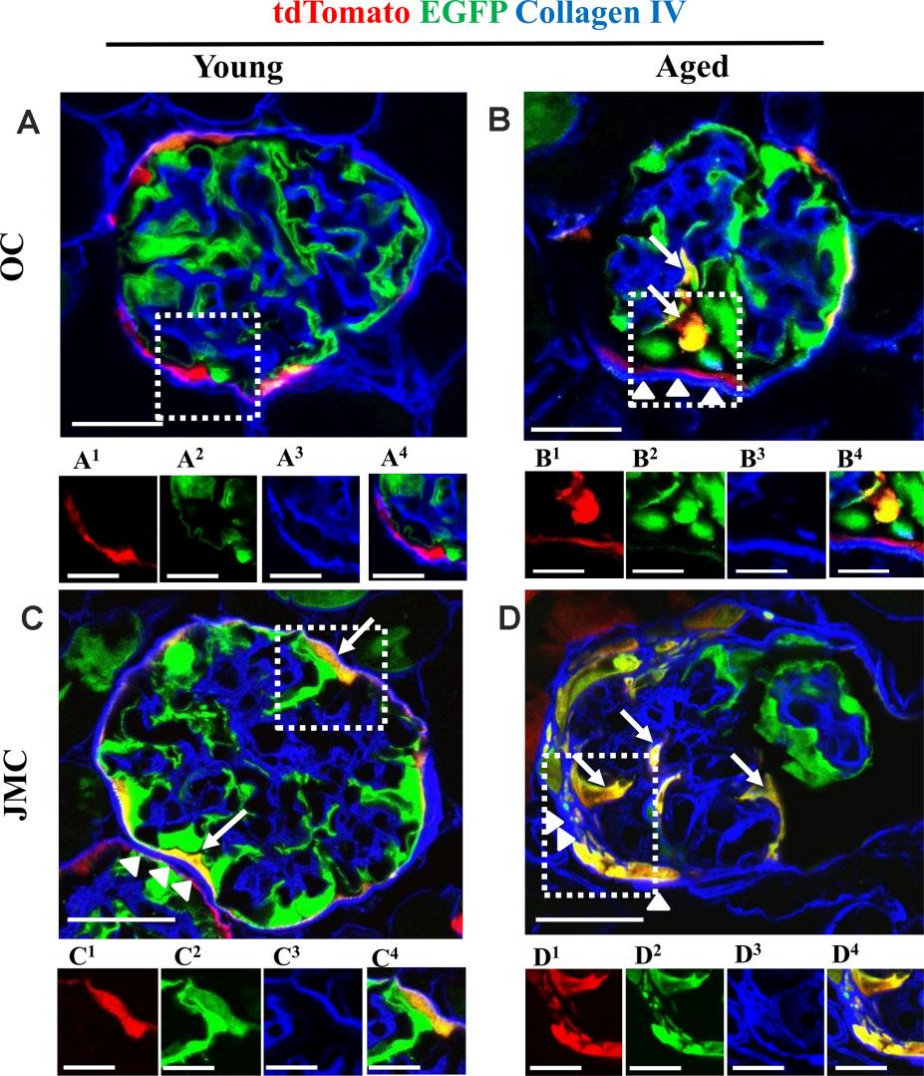
**Collagen IV staining increases along Bowman's capsule with accompanied migration of tdTomato^+^PECs in aged mice.** (**A**–**D**) Representative confocal images of tdTomato^+^ (red), EGFP^+^ (green) and Collagen IV staining (blue) in young and aged mice. The inserts show separate channels of the outlined glomeruli, with superscripts: 1=tdTomato, 2=EGFP, 3=Collagen IV and 4=merged. (**A**) Young mice (OC) showed that tdTomato^+^PECs are limited to Bowman’s capsule (**A^1^**) and EGFP^+^ podocytes to the glomerular tuft (**A^2^**). Collagen IV staining (blue) (**A^3^**) was used to delineate the kidney architecture. (**B**) Aged mice (OC) showed that differentiated yellow cells (tdTomato^+^PECs^+^ EGFP^+^) (marked with white arrows) were detected in the glomerular tuft (B^1^, B^2^) which was accompanied by an increase in Collagen IV staining along BC (white arrow heads) (**B^3^**). (**C**) Young mice (JMC) showed that differentiated yellow cells (tdTomato^+^PECs^+^ EGFP^+^) were detected in some glomeruli along Bowman’s capsule (**C^1^**, **C^2^**) which was accompanied by an increase in Collagen IV staining along BC (white arrow heads) (**C^3^**). (**D**) Aged mice (JMC) showed that differentiated yellow cells (tdTomato^+^PECs^+^ EGFP^+^) have migrated to the glomerular tuft (marked with white arrows) and co-express tdTomato (**D^1^**), EGFP (**D^2^**), which was accompanied by an increase in Collagen IV staining along BC (white arrow heads) (**D^3^**). Scale bars represent 25μm or 5μm (insets).

Co-staining with collagen IV (Col IV) was performed to determine extracellular matrix accumulation in aged glomeruli with PECs that differentiated towards a podocyte fate in the OC and JMC (Figure 4). There was an increase in collagen IV staining along Bowman’s capsule in aged OC glomeruli with newly generated podocytes from PEC origin compared to young mice (Figure 4A, 4B). In aged JMC glomeruli, the increased number of PECs that differentiated towards a podocyte fate was accompanied by a higher degree of collagen IV staining along Bowman’s capsule (Figure 4C, 4D).

These findings are similar to our previous report in a model of FSGS, where a subpopulation of PECs that differentiated towards a podocyte fate (TdTomato^+^/EGFP^+^ (yellow) cells) were observed along Bowman’s capsule in about 30.0% of young glomeruli [24]. In this study, aged mice showed a slightly higher percentage of glomeruli with differentiated PECs along Bowman’s capsule (40.0±7.0%). Previously, we described these cells as “parietal podocytes” or “transitional PECs” [32–35] and showed that they move to the glomerular tuft during FSGS [24], which we confirmed in this model of aging.

### Phenotypic and ultrastructural confirmation that a subset of PECs differentiate to a podocyte fate

**Figure 5:**
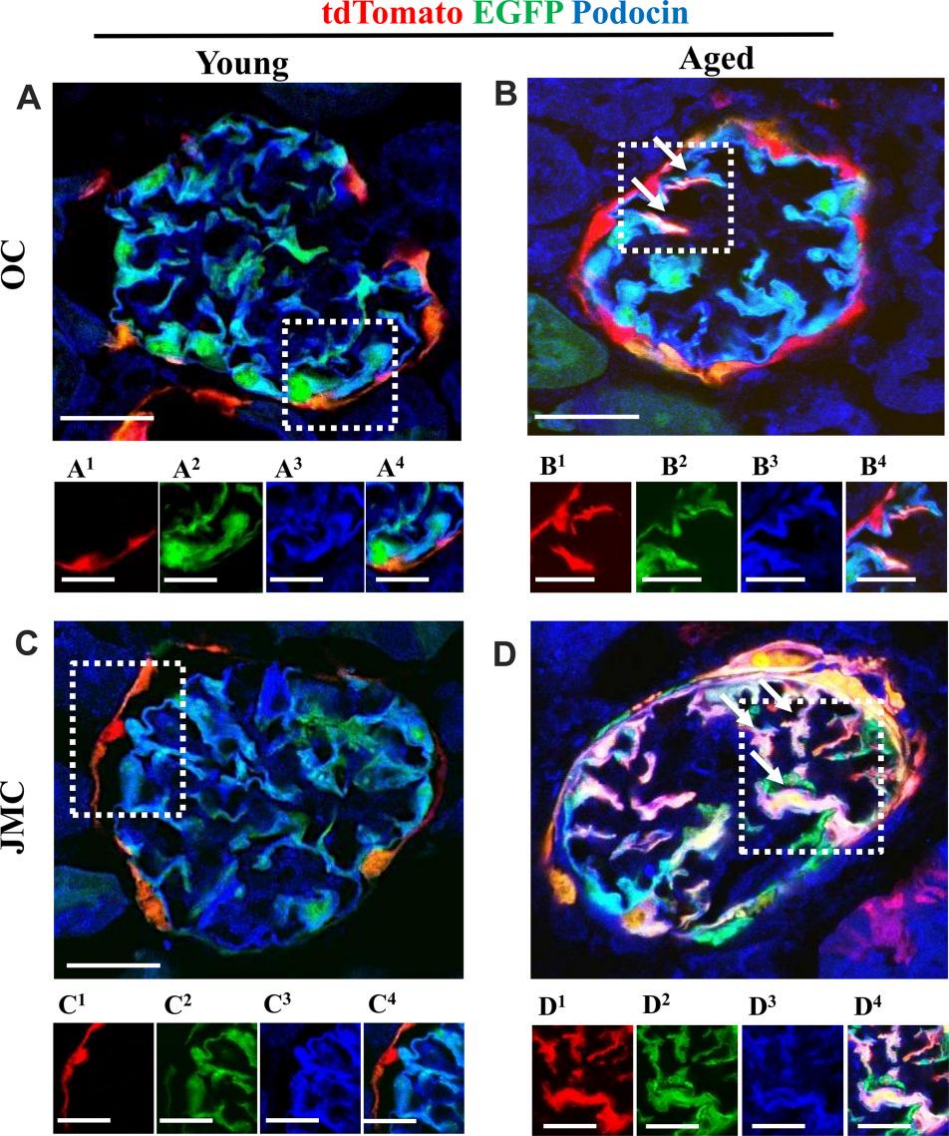
**A subset of newly generated podocytes** (**tdTomato^+^EGFP^+^**) **from PEC origin co-express podocin in the glomerular tuft of aged mice.** (**A**–**D**) Representative confocal images of tdTomato^+^ (red), EGFP^+^ (green) and podocin staining (blue) in young and aged mice. The inserts show separate channels of the outlined glomeruli, with superscripts: 1=tdTomato, 2=EGFP, 3=podocin and 4=merged. Podocin staining was detected with an antibody, tdTomato and EGFP reporters were detected without antibody. (**A**) Young mice (OC) showed that tdTomato^+^PECs (red) are detected along Bowman’s capsule (**A^1^**). The majority of EGFP^+^ cells (green) (**A^2^**) co-localize with podocin (blue) (**A^3^**) and create a cyan color (**A^4^**). (**B**) Aged mice (OC) showed that a subset of differentiated tdTomato^+^ PECs (red) (**B^1^**) co-expresses EGFP^+^ (green) (**B^2^**) and podocin (blue) (**B^3^**) creating a pink/white color in the glomerular tuft (white arrows) (**B^4^**). (**C**) Young mice (JMC) showed that tdTomato^+^PECs (red) (**C^1^**) are detected along Bowman’s capsule. The majority of EGFP^+^ cells (green) (**C^2^**) co-localize with podocin (blue) (**C^3^**) in the glomerular tuft creating a cyan color (**C^4^**). (**D**) Aged mice (JMC) showed that a higher number of tdTomato^+^PECs (red) (**D^1^**) have migrated to the glomerular tuft (marked with white arrows), become EGFP^+^ (**D^2^**) and co-localized with podocin (blue) (**D^3^**), creating a pink/white color (**D^4^**). Scale bars represent 25μm or 5μm (insets).

**Figure 6:**
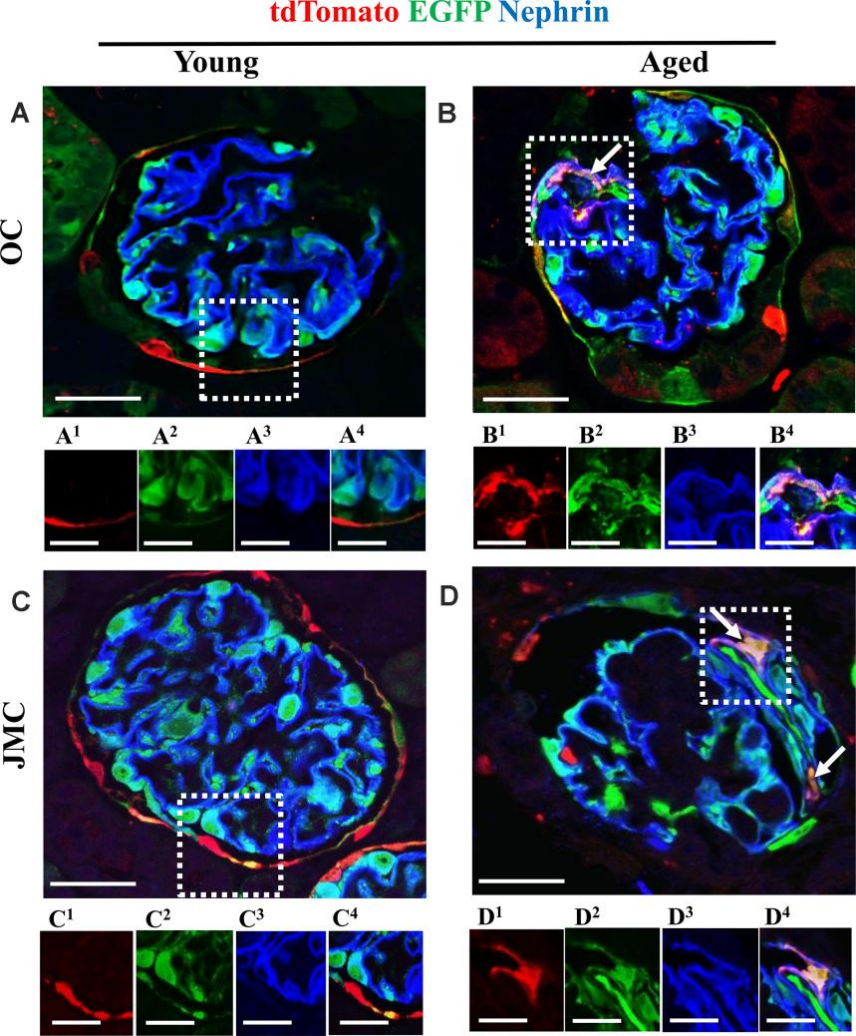
**A subset of newly generated podocytes from PEC origin** (**tdTomato^+^EGFP^+^**) **co-express nephrin in the glomerular tuft of aged mice.** (**A**–**D**) Representative confocal images of tdTomato staining (red fluorescent protein, RFP) (red), EGFP^+^ (green) and nephrin staining (blue) in young and aged mice. The inserts show separate channels of the outlined glomeruli, with superscripts: 1=tdTomato, 2=EGFP, 3=nephrin and 4=merged. (**A**) Young mice (OC) showed that tdTomato^+^PECs (red) were observed along Bowman’s capsule (**A^1^**) and genetically labeled podocytes (EGFP^+^ cells, green) (**A^2^**) co-express nephrin (blue) (**A^3^**), creating a cyan color (**A^4^**). (**B**) Aged mice (OC) showed that a subset of tdTomato^+^ PECs (labeled with red fluorescent anti-RFP) (**B^1^**) co-expresses EGFP^+^ (green) (**B^2^**) and nephrin (blue) (**B^3^**) creating a pink/yellow color in glomerular tuft (marked with white arrow) (**B^4^**). (**C**) Young mice (JMC) showed that tdTomato^+^ staining PECs were observed along Bowman’s capsule (**C^1^**) EGFP staining (**C^2^**) was detected in a typical podocyte distribution and overlaps with nephrin staining (**C^3^**), creating a cyan color (**C^4^**). (**D**) Aged mice (JMC) showed that tdTomato^+^ staining PECs (**D^1^**) (marked with white arrows) have migrated onto the glomerular tuft and differentiated to a podocyte fate, co-staining with EGFP (green) (**D^2^**) and nephrin (blue) (**D^3^**) creating pink/yellow color (**D^4^**). Scale bars represent 25μm or 5μm (insets).

To prove that a subset of labeled PECs that migrated to the glomerular tuft acquired podocyte-like features, immunofluorescence staining was performed for podocin (Figure 5) and nephrin (Figure 6). Antibodies were not required for detection of tdTomato reporter-labeled PECs and EGFP reporter-labeled podocytes. There was no overlap between the three colors in young mice in OC and JMC glomeruli. In contrast, cells with a white/purple color, representing the merge of red (tdTomato), green (EGFP) and blue (used for podocin and nephrin) were detected in a subset of both OC and JMC glomeruli of aged mice. In contrast, podocytes derived from PECs (yellow cells) did not co-express Perlecan (Supplementary Figure 3), used as a mesangial cell marker, nor CD31, used as an endothelial cell marker (Supplementary Figure 4).

Immunofluorescence staining with anti-RFP (tdTomato PEC reporter) and anti-EGFP (podocyte reporter) on paraffin embedded kidney sections from young, middle age and aged mice, with sequential peroxidase staining, detecting p57 staining on the same sections, showed that the majority of p57^+^ cells were EGFP labeled in young mice (Supplementary Figure 2A and 2B). A subpopulation of anti-RFP labeled cells (tdTomato^+^ PECs) observed in the glomerular tuft of middle age mice were p57-positive. (Supplementary Figure 2C, 2D) Newly generated podocytes from PEC origin observed in glomeruli of aged mice were anti-RFP-positive, anti-EGFP-positive and p57-positive (Supplementary Figure 2E, 2F).

**Figure 7:**
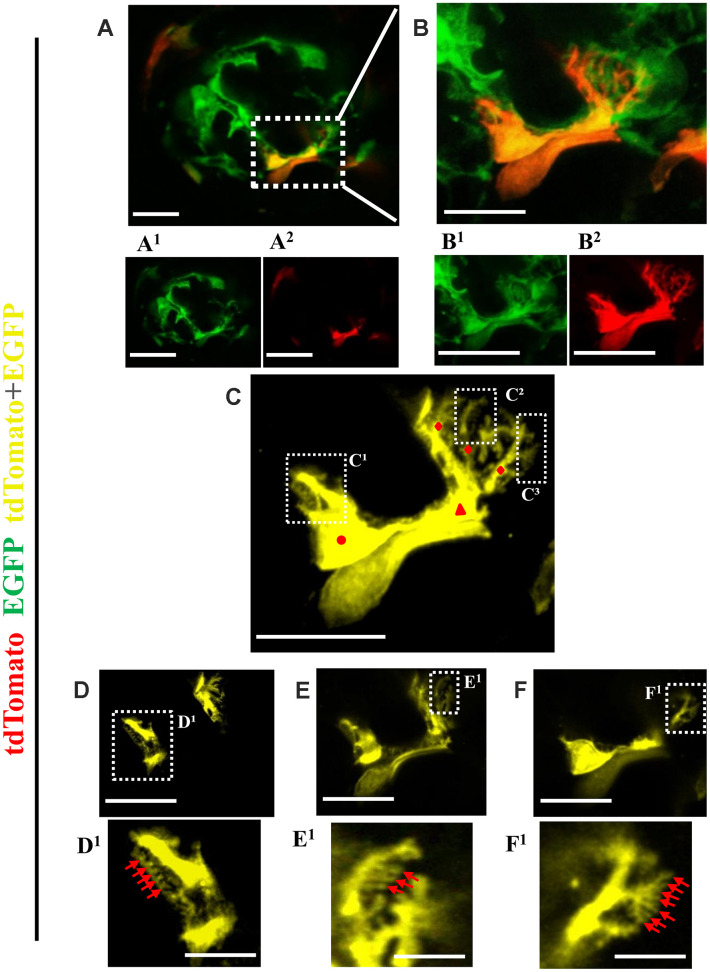
**Ultrastructure of newly generated podocytes from PEC origin** (**tdTomato^+^EGFP^+^**) **in the glomerular tuft of aged mice.** (**A**) Representative confocal image of expansion microscopy of a glomerulus in an aged mouse with a newly generated podocyte from PEC origin (marked with dashed box). Superscripts show separate channels: tdTomato^+^ (red) (**A^1^**) and EGFP^+^ (**A^2^**). (**B**) Higher magnification of image shown in A of newly generated podocyte, with accompanied single color images of tdTomato (**B^1^**) and EGFP (**B^2^**). (**C**) The newly generated podocyte has classic podocyte architecture: a cell body (labeled with red circle), primary process (red triangle), several secondary processes (red rhombuses), branching to minor and tertiary foot processes (**C^1^**–**C^3^** labeled with dashed boxes). (**D**–**F**) Higher magnification of images shown in dashed boxes **C^1^**–**C^3^** with different z-stack focal planes show various tertiary foot processes (labeled with red arrows). Scale bars represent 25μm or 5 μm (inset) and are in pre-expansion dimensions.

Finally, expansion microscopy was performed to define ultrastructure (Figure 7). Single channels show that the green cell (Figure 7A^1^ and 7B^1^) on the glomerular tuft was derived from PEC origin (red color) (Figure 7A^2^ and 7B^2^) to create a yellow color with major, primary and secondary foot processes (Figure 7C). Figures 7D, E and F show consecutive z-stacks through the newly generated podocyte and higher magnified images show tertiary foot processes (Figure 7D^1^, 7E^1^, 7F^1^).

Taken together, in individual glomeruli typified by podocyte depletion, a subset of cells of PEC origin migrate to the glomerular tuft and de novo express three podocyte proteins, and acquire ultrastructural features characteristic of podocytes.

### Podocyte-like cells derived from PECs express de novo VEGF-A

**Figure 8:**
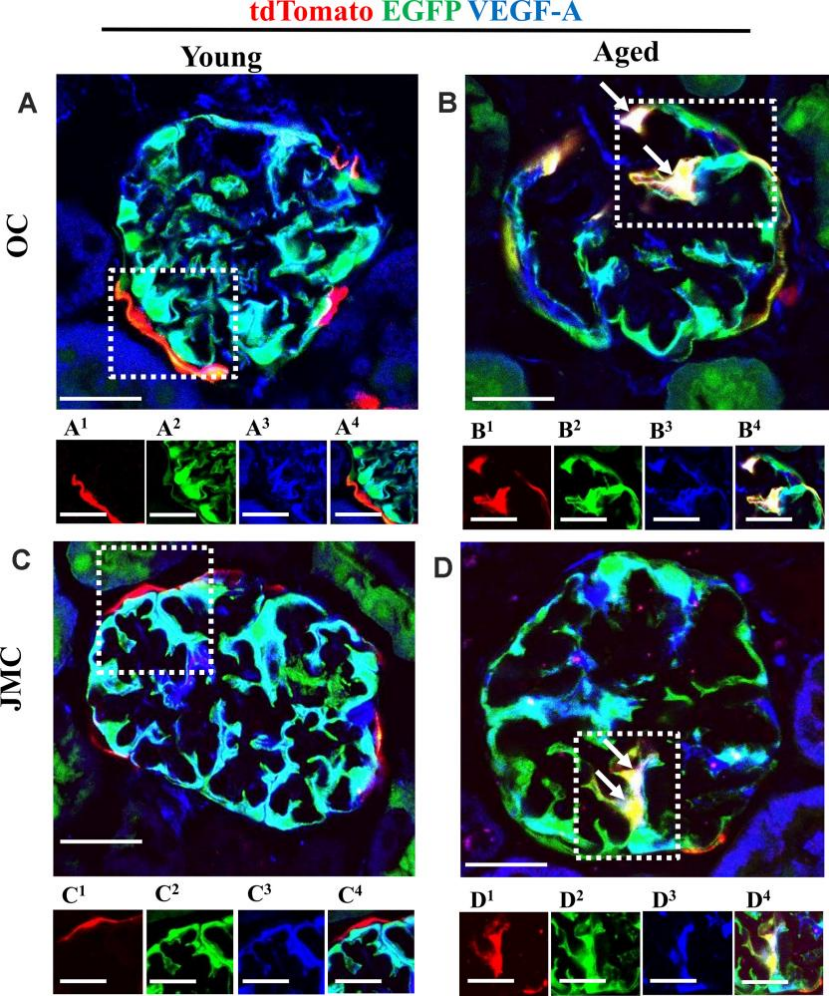
**A subset of newly generated podocytes from PEC origin** (**tdTomato^+^EGFP^+^**) **de novo express VEGF-A in the glomerular tuft of aged mice.** (**A**–**D**) Representative confocal images of tdTomato^+^ (red), EGFP^+^ (green) and VEGF-A (blue, podocyte specific marker) in young and aged mice. The inserts show separate channels of the outlined glomeruli, with superscripts: 1=tdTomato, 2=EGFP, 3=VEGF-A and 4=merged. (**A**) Young mice (OC) showed that tdTomato^+^PECs were observed along Bowman’s capsule (**A^1^**). EGFP reporter (**A^2^**) overlaps with VEGF-A staining (**A^3^**) and creates a cyan color (**A^4^**). (**B**) Aged mice (OC) showed that a subset of differentiated tdTomato^+^ PECs (red) (**B^1^**) co-expresses EGFP^+^ (green) (**B^2^**) and VEGF-A (blue) (**B^3^**) creating a yellow/white color in the glomerular tuft (white arrows) (**B^4^**). (**C**) Young mice (JMC) showed that tdTomato^+^ staining PECs were observed along Bowman’s capsule (**C^1^**) EGFP staining (**C^2^**) was detected in a typical podocyte distribution and overlaps with VEGF-A staining (**C^3^**), creating a cyan color (**C^4^**). (**D**) Aged mice (JMC) showed that tdTomato^+^ staining PECs (**D^1^**) (marked with white arrows) have migrated onto the glomerular tuft and differentiated to a podocyte fate, co-staining with EGFP (green) (**D^2^**) and VEGF-A (blue) (**D^3^**) creating pink/yellow color (**D^4^**). Scale bars represent 25μm or 5μm (insets).

Within the glomerulus, VEGF is considered podocyte-derived [24, 36–38]. Figure 8 shows VEGF-A staining localized to podocytes in young mice (Figure 8A and 8C). In aged mice, a subset of newly generated podocytes derived from PECs express VEGF-A, creating a white/yellow color, in both OC and JMC glomeruli (Figure 8B and 8D). This suggests that these cells acquire one of the synthetic functions typified by podocytes.

### Subpopulations of migrated PECs co-express CD44, but newly generated podocytes from PEC origin do not express PEC the marker PAX8 and do not proliferate in the glomerular tuft of aged mice

**Figure 9:**
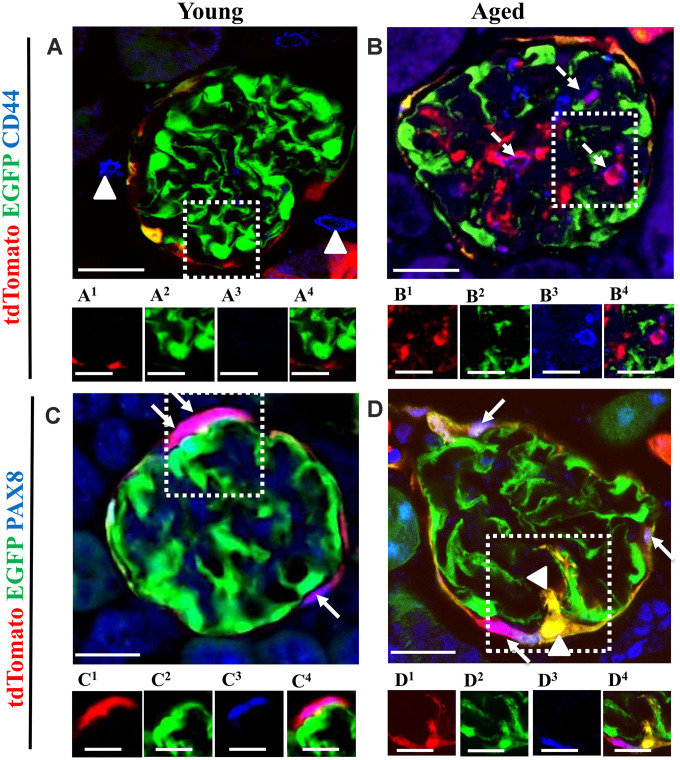
**The majority of tdTomato^+^PECs co-express PAX8 along Bowman’s capsule, however newly generated podocytes from PEC origin no longer express PAX8. Activated PECs** (**tdTomato^+^CD44^+^**) **were detected in the glomerular tuft of aged mice.** (**A**, **B**) Representative confocal images of tdTomato^+^ (red), EGFP^+^ (green) and CD44^+^ (blue) in young and aged mice. The inserts show separate channels of the outlined glomeruli, with superscripts: 1=tdTomato, 2=EGFP, 3=CD44 and 4=merged. (**A**) Young mice showed that CD44 staining was not detected in the glomerular tuft. An occasional CD44^+^ cell was observed around glomeruli (marked with arrow heads). Each channel for the glomerular tuft region marked with the dashed box are presented in panels (**A^1^**–**A^4^**). (**B**) Aged mice showed that a subpopulation of tdTomato^+^PECs (**B^1^**) that migrated to glomerular tuft were positive for CD44 (magenta color, B^3^- B^4^), marked with dashed arrows, with no overlap with EGFP (**B^2^**). (**C**, **D**) Representative confocal images of tdTomato^+^ (red), EGFP^+^ (green) and PAX8 (blue) in young and aged mice. The inserts show separate channels of the outlined glomeruli, with superscripts: 1=tdTomato, 2=EGFP, 3=PAX8 and 4=merged. (**C**) Young mice showed that PAX8 staining (**C^3^**) was detected in PECs along Bowman’s capsule and co-localized with tdTomato reporter (**C^1^**) creating a pink/purple color (**C^4^**) (marked with white arrows). PAX8 was not detected in podocytes (EGFP^+^ cells) (**C^2^**). (**D**) Aged mice showed that the majority of tdTomato^+^PECs (**D^1^**) co-express PAX8 (**D^3^**) creating a magenta/white color (**D^4^**) (marked with solid arrow). However, newly generated podocytes from PEC origin (tdTomato^+^EGFP^+^) (**D^1^**, **D^2^**) do not express PAX8 in the glomerular tuft of aged mice (marked with arrow heads). Scale bars represent 25μm or 5μm (insets).

CD44 is a marker of PEC activation, defined as a pro-fibrotic and migratory phenotype, and increases in aged PECs [3, 19, 39]. However, it is also a marker for progenitors in certain non-kidney cells [40–42]. CD44 was not detected in the glomeruli of young mice (Figure 9A). However, CD44 was detected in a subset of PECs along Bowman’s capsule and PECs that migrated to the glomerular tuft (Figure 9B).

PECs were identified by PAX8^+^ staining and the majority of tdTomato^+^ cells co-localized with PAX8 along Bowman’s capsule of young mice (Figure 9C). However, newly generated podocytes from PEC origin do not express PAX8 in the glomerular tuft in aged mice (Figure 9D).

In order to measure proliferation in aged mice, staining for Ki67 was used [43]. Ki67^+^ cells were detected in the interstitium in young mice (OC and JMC) (Supplementary Figure 5A, 5C). Likewise, occasional tubular cells expressed Ki67 in the OC compartment in aged mice (Supplementary Figure 5B). A subpopulation of PECs expressed Ki67 in aged JMC glomeruli, but did not express EGFP (Supplementary Figure 5D). These results indicate, that a subset of activated PECs migrate to glomerular tuft but newly generated podocytes from PEC origin do not express PAX8 and do not proliferate in aged glomeruli.

## DISCUSSION

Healthy aging is accompanied by changes to the kidney, including a progressive decline in podocyte number and density [5, 22]. Because terminally differentiated podocytes are unable to self-renew, any replacement has to derive from another cell source, namely a podocyte progenitor [9, 44]. In the current study, we used a genetic approach in a dual PEC-PODO reporter mouse [24] to ask if glomerular parietal epithelial cells (PECs) can indeed differentiate to a podocyte fate as podocyte number progressively declines with advancing age. Our results show that in glomeruli in which podocyte number declines below a certain threshold, a subset of PECs migrate to the glomerular tuft, where they de novo express podocyte markers, make VEGF-A and acquire podocyte ultrastructural features.

The regeneration of podocytes is an active area of study, because when these terminally differentiated epithelial cells decrease in number in disease, or in healthy aging, they are unable to proliferate, and thus cannot self-renew. Reduced podocyte number underlies glomerular scarring [45]. Thus, any replacement of “lost” podocytes must derive from another cell source. PECs have been considered a progenitor source for both adolescent podocytes during normal kidney maturation [46], and adult podocytes in disease [10–12]. However, some studies in adult mice have refuted this notion [18, 46]. Several considerations might underlie the differences in results, including mouse species, the use of reporter labeling, the cell specific gene used for reporting, mouse sex and age, the type of experimental animal models used, methodologies used to assess podocyte number, and others.

Why is the methodology to measure podocyte number important in this debate? [47–49] Methods that rely on the podocyte mean derived by summing podocyte number (numerator) in a fixed number of glomeruli (denominator) regardless of their degree of injury, will underestimate podocyte replacement in individual glomeruli because of the focal nature of disease in healthy aging. In the current study, we make two important observations in this context. First, we noted that in healthy glomeruli (defined as no injury on PAS or silver staining) of middle-aged and aged mice, podocyte number was typically normal. In contrast, with increasing glomerular injury with advancing age, podocyte number decreased in those individual glomeruli with injury scores of 1, 2 and 3. Second, within individual glomeruli with a normal podocyte number per glomerular cross section, no labeled PECs migrated to the tuft. This makes sense from a regeneration viewpoint, as there is no demand for regeneration in the setting of a normal podocyte number. However, within individual glomeruli, there is a threshold of podocyte number (less than 10 per glomerular cross section) that correlates with the migration of (red) PECs to the tuft, and a second threshold that accompanies the subsequent differentiation of PECs to a podocyte fate (less than 7 per glomerular cross section), evidenced by a yellow color (Figure 3). Moreover, as podocyte number decreases further in individual glomeruli, both PEC migration to the glomerular tuft and differentiation to a podocyte fate increases. There is no differentiation in glomeruli with a normal podocyte number, because there is no need.

This is significant, because although the overall percentage of glomerular cross sections with red and yellow cells was only 4.1% and 1.7% respectively, in terms of accessing possible repair, we should consider glomeruli where repair is warranted, because a glomerulus with a healthy podocyte population would have no need for replacement of cells. In glomeruli with podocyte loss that was great enough to warrant migration (red cells on the tuft), 41.0% of those glomeruli experienced differentiation to a podocyte fate, defined by the presence of a yellow cell. The majority of yellow cells had phenotypic features of podocytes (see below).

We recognize that a potential pitfall of this analysis is only considering glomerular cross sections that are 4μm thick, in the context that glomeruli are ~ 70 μm in diameter. Moreover, the area of the tuft was not taken into consideration when assessing p57. If one assumes that a glomerular tuft is a sphere with equally distributed podocytes, the best-case scenario of a slice through the exact center, we are sampling only 8.6% of each glomerulus. However, despite this relatively small sampling per glomerulus, and the fact that PEC number decreases with advancing age, observing events of migration and differentiation suggest they are actually not rare events, and are plausibly biologically relevant. Another pitfall in the underestimation of migration and differentiation of PECs is that the overall PEC number decreases with advancing age [19, 20]. That said, the combination of reduced PEC progenitor number with age, and many of the remaining PECs undergoing changes such as to a myofibroblast phenotype, reduce the biological plausibility that robust differentiation of PECs to a podocyte fate decreases progressively with advancing age, and therefore may not be adequate for full regeneration and repair.

We acknowledge weaknesses in these studies. First, we have not accounted for changes to the immune system with aging, both locally in the kidney, and systemically. Second, mouse strain might influence outcomes. Third, sex differences need to be better understood as a potential confounder for podocyte regeneration. Finally, we have not determined if any pharmacological interventions could either enhance podocyte regeneration in aged mice, or even prevent age-related changes to podocytes. That said, we have previously reported that PEC number is higher in aged mice given the mitochondrial stabilizer SS-31 [3] or the mTOR regulator rapamycin [39], raising the possibility that in doing so, the reservoir of PEC progenitors is increased. Further studies are needed in aged dual PEC-Podo mice.

The following results support the notion that a subset of PECs do indeed differentiate to a podocyte fate in aged glomeruli: yellow cells acquire de novo expression of podocin, nephrin, p57 and VEGF-A, and change morphology to a cell with primary, secondary and tertiary processes. These cells do not express CD44, which reinforces the paradigm that when CD44 is increased in PECs, this is not a regenerative transition, but rather one that likely reduces their capacity to differentiate to a podocyte fate.

We have previously reported that with advancing age, glomeruli in the juxta-medullary portion of the cortex are more severely injured than those in the outer cortex [3, 19, 20]. Accordingly, we asked if the region of glomeruli within the kidney impacts PEC differentiation to a podocyte fate, by examining individual OC and JMC glomeruli. The results show that, the percentage of differentiation occurring on a yes/no basis per glomerulus was 38.5% in OC glomeruli and 44.7% in JMC glomeruli.

Finally, one should ask if the replacement of podocytes in individual glomeruli by PEC progenitors is sufficient in the aged kidney? While podocyte replacement clearly derives from PEC origin in a subset of glomeruli, this is not a universal phenomenon. Overall, podocyte number is lower in healthy aged mice than in healthy young and middle-aged mice. Taken together, we conclude from the data in this study that the capacity for PEC progenitors to transition to a podocyte fate is reduced with aging, compared to younger mice. We speculate that this is multi-factorial, including a decrease in the number of PEC progenitors and changes to their phenotype from an epithelial state to a myofibroblast-like state.

## MATERIALS AND METHODS

### Animals

Dual reporting *PEC-rtTA|LC1|tdTomato*|*Nphs1-FLPo*|*FRT-EGFP* (named PEC-PODO mice in this manuscript) were bred by first crossing previously reported PEC-reporter mice [15, 46, 50, 51], with the tdTomato clone Ai14 mouse (The Jackson Laboratory Stock #007914) [52] to replace the original Rosa26 (LacZ) reporter. These PEC|tdTomato reporter mice were then crossed with our previously generated *Nphs1-FLPo|FRT-EGFP* mouse. The *Nphs1-FLPo|FRT-EGFP* mouse expresses FLP recombinase as previously described by Goldberg et. al. [53], resulting in the FLP mediated excision of an FRT flanked STOP in the RCE:FRT [54] (JAX Stock#: 010812) mouse available from The Jackson Laboratory (Bar Harbor, ME). Resulting animals have conditional tdTomato expressing PECs upon administration of doxycycline, and constitutive EGFP expression in podocytes. Control mice comprised of quadruple transgenic mice without the rtTA or FLPo. Mice were housed in the animal care facility of the University of Washington under specific pathogen-free conditions. Studies were reviewed and approved by the University of Washington Institutional Animal Care and Use Committee (2968-04).

### Reporter induction

Adult mice (10-12 weeks of age) were fed ad libitum 625mg/kg Doxycycline Chow (TD.01306, Envigo, Indianapolis, IN) for 3 weeks to induce permanent labeling of PECs with tdTomato reporter. Animals were otherwise provided standard housing, ad libitum food and water. No drugs were required for the induction of the EGFP reporter.

### Tissue collection

Male and female mice were randomly divided into two cohorts. One cohort was terminally sacrificed at 6 months of age (young), while the second were designated for aging. Animals designated for aging underwent survival biopsies at 14 months of age (middle age) as previously reported [20] under aseptic conditions as approved by the University of Washington Institutional Animal Care and Use Committee. For terminal kidney necropsies at 6 months of age (n=9) or 20-24 months of age (aged, n=14), mice were killed with an overdose of Ketamine (JHP Pharmaceuticals LLC, Rochester, MI)/Xylazine (Patterson Veterinary, Devens, MA)as previously described [27]. Kidney tissue was butterflied and one half butterfly was placed into 4% paraformaldehyde (PFA, Affymetrix, Santa Clara, CA) in PBS for 45 minutes, washed briefly in PBS, placed in 30% sucrose (Sigma-Aldrich, St Louis, MO) in PBS overnight, blotted dry, embedded in Tissue-Tek® O.C.T. Compound (VWR, Radnor, PA), and frozen in a 100% ethanol/dry ice bath. The second half of the butterflies were placed into 10% neutral buffered formalin (NBF) (Globe Scientific, Mahwah, NJ) overnight at 4°C, transferred to 70% ethanol, and embedded in paraffin.

### Assessment of Glomerulosclerosis and Podocyte Depletion

Immunostaining was performed for p57 with PAS counterstaining to measure podocyte density and assess glomerulosclerosis, as previously reported [34, 48, 55, 56]. In brief, paraffin sections were de-paraffinized and rehydrated. Antigen retrieval was performed by microwave heating in 1 mM EDTA, pH 8.0 for 10 min. The following solutions were applied for blocking: 3% hydrogen peroxide for 20 minutes and freshly made 5% nonfat dry milk for 30 minutes. Secondary horseradish peroxidase conjugated antibodies were detected with diaminobenzidine (DAB, Sigma-Aldrich). Counterstaining was performed with periodic-acid Schiff and hematoxylin as previously described [39]. Slides were dehydrated in ethanol and mounted with Histomount (National Diagnostics, Atlanta, GA). Kidney cortex was divided in two compartments: outer cortex (OC) and juxta-medullary cortex (JMC) as previously described [3, 20]. An average of 45±10 glomeruli from the OC and 20±5 glomeruli from the JMC were quantified for each mouse. Every captured glomerulus was ranked on a scale of 0-3 to determine glomerulosclerosis as previously described [3]. In brief: 0 = no injury, 1 = one third of the glomerular tuft cross-section showed mesangial and limited or partial Bowman’s capsule thickening; 2 = greater than 50% of the glomerular tuft showed mesangial and Bowman’s capsule thickening, loss of podocytes and capillary loop structure; 3 = entirely sclerotic glomerulus. For each compartment (OC and JMC) the total mean score was calculated as previously described [3].

### Assessment of glomerular scarring and extracellular matrix

Jones’ basement membrane staining was performed by the University of Washington Pathology Research Services Laboratory on tissue embedded in paraffin from young, middle age and aged mice in order to determine extracellular matrix accumulation.

### Multicolor immunofluorescence staining

Immunostaining was performed on 4μm sections from frozen and formalin fixed paraffin-embedded (FFPE) tissue as previously described [57]. Briefly, frozen sections were allowed to air-dry and OCT compound was removed in PBS (pH 7.4). Paraffin sections were deparaffinized and blocked for biotin (Vector Labs, Burlingame, CA). Background buster (Accurate Chemical & Scientific, Westbury, NY) was used to block non-specific antibody binding. The appropriate biotinylated secondary antibody (Vector Laboratories) was applied followed by Streptavidin, AlexaFluor 647 conjugate (Life Technologies - Molecular Probes, Grand Island, NY). All immunofluorescence samples were mounted using Vectashield with DAPI (Vector Labs, Burlingame, CA). As a negative control, staining was performed without primary antibody.

### Primary antibodies and conditions for immunofluorescence

In order to visualize tdTomato and EGFP on FFPE samples, the following antibodies were applied: DyLite 594 conjugated red fluorescent protein antibody (1:100, Rockland Inc. Limerick, PA), that specifically detects the tdTomato reporter and anti-EGFP green fluorescent protein (1:100, Clontech, Mountain View, CA). To identify podocytes, the following antibodies were used: anti-podocin (1:4,000, Abcam, ab50339, Cambridge, MA), anti-nephrin (1:500, Fitzgerald Industries International. Inc., Concord, MA) and anti-p57 (1:500, Santa Cruz Biotechnology, Dallas, TX). To outline the kidney architecture, biotinylated collagen IV antibody (1:100, Southern Biotechnology, 1340-08, Birmingham, AL) was applied overnight. Kidney endothelial cells were marked by a CD31 antibody (1:100, Abcam, Cambridge, MA). “Activated” PECs were marked by staining with a CD44 antibody (1:50, Clone IM7, BD Biosciences, San Jose, CA). Mesangial cells were identified by using a Perlecan antibody (gift from Jeff Miner). To determine vascular growth factor expression, which is highly expressed in podocytes [24, 36–38], staining with VEGF-A antibody (1:200, Abcam, ab 52917, Cambridge, MA) was performed overnight. To identify parietal epithelial cells (PECs) immunostaining with a Pax8 antibody (1:500, Protein Tech Group, Chicago, IL) was performed overnight. To detect proliferation in aged dual reporter mice, a Ki67 [43] antibody was used (1:100, Thermo Fisher Scientific, Waltham, MA, USA). The appropriate biotinylated secondary antibody (1:400, Vector Laboratories, Burlingame, CA) was applied, followed by Streptavidin AlexaFluor 647 conjugate (1:200, Life Technologies - Molecular Probes, Grand Island, NY).

### Multicolor immunofluorescence by sequential peroxidase staining

Paraffin sections were deparaffinized and rehydrated. Antigen retrieval was performed by microwave heating in 1 mM EDTA, pH 6.0 for 10 min. The following solutions were applied for blocking: Avidin/Biotin solutions (Vector Laboratories, Burlingame, CA) for 20 minutes and background buster (Accurate Chemical & Scientific, Westbury, NY) for 30 minutes. The following primary antibodies were applied overnight to detect the reporters: Anti-RFP (tdTomato reporter) (1:100, Rockland Inc. Limerick, PA) and anti-EGFP (podocyte specific reporter) (1:100, Clontech, Mountain View, CA). This was followed by the appropriate biotinylated secondary antibody (Vector Laboratories, Burlingame, CA) and then by Streptavidin, AlexaFluor 488 conjugate (1:200, Life Technologies - Molecular Probes, Grand Island, NY). Slides were mounted with Vectashield/DAPI mounting media (Vector Laboratories, Burlingame, CA). Fluorescent images were collected at 400x magnification. Coverslips were gently removed by soaking slides in PBS, and slides were subsequently stained for p57/PAS as described above. Brightfield images were collected and combined with immunofluorescent images, showing the same glomeruli in both sets of images.

### Quantification of migrated PECs to glomerular tuft

Migrated PECs were identified as cells expressing TdTomato^+^ or TdTomato^+^ / EGFP^+^ reporters in the glomerular tuft area. Young mice: An average of 1115 glomeruli were sampled, of which 951 (85.3%) were OC glomeruli and 164 (14.7%) were JMC glomeruli. Middle aged: An average of 533 glomeruli were sampled, comprising 473 (88.7%) in the OC and 60 (11.2%) in the JMC. Aged mice: An average of 1605 glomeruli were sampled, of which 1384 (86.2%) were OC and 221 (13.8%) were JMC glomeruli respectively.

### Quantification of podocytes in glomeruli with PECs migrated to the glomerular tuft

Three color staining for p57 and the RFP and EGFP reporters was performed, and the number of p57^+^ cells in the OC and JMC were measured for each animal. Data were expressed as the average number of p57^+^ cells per kidney cross section.

### Expansion of a kidney specimen using ExM

Expansion microscopy was performed by using a previously described protocol [58]. Dual reporting PEC-PODO mice (n=2) were sacrificed at 20-24 months of age as described above. Murine kidneys were fixed with 4% paraformaldehyde (PFA, Affymetrix, Santa Clara, CA) in PBS for 45 minutes and incubated overnight in 30% sucrose (Sigma-Aldrich, St Louis, MO) in PBS. They were then washed with PBS and cut into 60 μm thick slices. The slices were incubated with MA-NHS in PBS for 1 hour at room temperature followed by three washes with PBS. Gelation solution was prepared by mixing: monomer solution (1× PBS, 2 M NaCl, 2.5% (wt/wt) acrylamide, 0.15% (wt/wt) N,N′-methylenebisacrylamide, 8.625% (wt/wt) sodium acrylate) and was applied for 1 hour at 4 °C, followed by incubation with monomer 0.01% (wt/wt) TEMPO solution (45 minutes). Lastly, TEMPO in monomer solution to final concentration of 0.2% (wt/wt) with ammonium persulfate (APS) added was immediately applied to the tissue, and a coverslip was placed on top. Tissues were allowed to gel at 37 °C for 1.5–2.5 h. in a humidified chamber. Tissue digestion was performed overnight at 37 °C with proteinase K digestion buffer (1× TAE buffer, 0.5% Triton X-100, 0.8 M guanidine HCl, 8 units mL^−1^ proteinase K), followed by washing with PBS. Next, kidneys were digested in collagenase buffer (1× HBSS, 0.7 mM CaCl_2_, 5 mg mL^−1^ collagenase) overnight at 37 °C. Tissue expansion was performed by incubating kidney slices in DI water for 30 min with 1–2 exchanges. Poly-L-lysine-coated coverslips were used for mounting. Imaging was then immediately performed on the samples.

### Microscopy

Two–dimensional images were detected on a Leica TCS SPE II laser scanning confocal microscope (Leica, Wetzlar, Germany) using 400x magnification and an EVOS FL Cell imaging system (Thermo Fisher Scientific, Waltham, MA, USA) using 200x magnification.

### Statistical analysis

Data are shown as the mean ± S.E.M. Student’s t-test was applied for comparisons between groups. Multiple groups were compared using one-way ANOVA with post hoc Tukey HSD test. P values <0.05 represented statistically significant differences.

## Supplementary Material

Supplementary Figures
