# Supplementary material for: Parietal epithelial cell differentiation to a podocyte fate in the aged mouse kidney
Source: Aging (Albany NY). 2020 Aug 28;12(17):17601–24. doi: 10.18632/aging.103788 (PMC7521511; doi:10.18632/aging.103788)
Supplement: Supplementary Figures [file aging-12-103788-s001..pdf]

SUPPLEMENTARY FIGURES

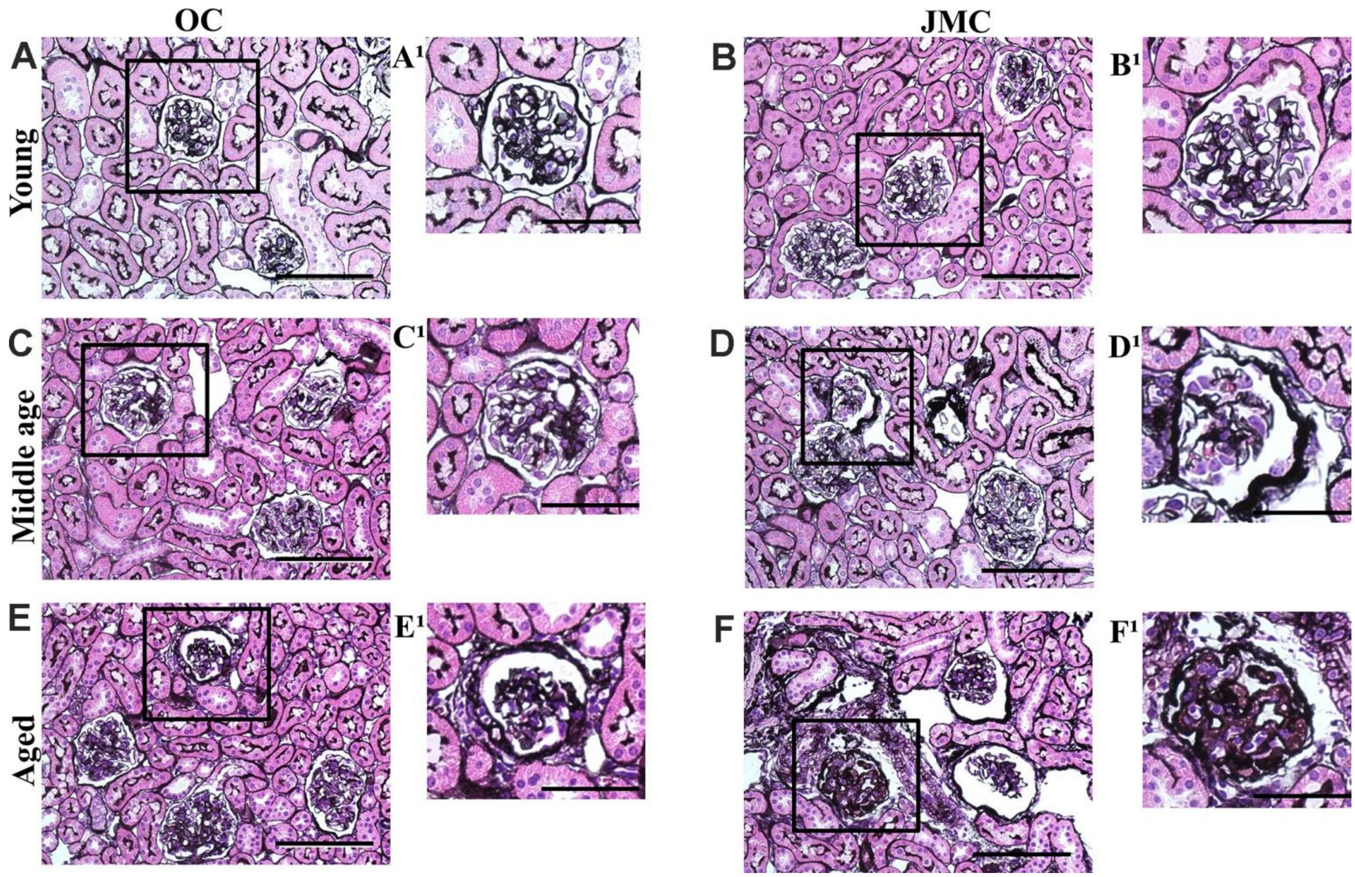

Supplementary Figure 1. Glomerular scarring and extracellular matrix accumulation in basement membrane was higher in aged mice. (**A**–**F**) Representative images taken at 20x of Jones' (Silver) staining in young (**A**, **B**), middle age (**C**, **D**) and aged mice (**E**, **F**). Glomeruli were divided into outer cortex (OC) (**A**, **C**, **E**) and juxta-medullary cortex (JMC) (**B**, **D**, **F**) compartments. Small inserts (40x) represent examples of glomeruli from each group, (**A^1**, **B^1**)-young mice, (**C^1**, **D^1**)-middle age glomeruli (OC and JMC) with increased thickness of BC (**E^1**)-OC aged glomeruli with segmental sclerosis and increased thickness of BC, (**F^1**)- JMC aged glomeruli with severe sclerosis and increased thickness of BC.

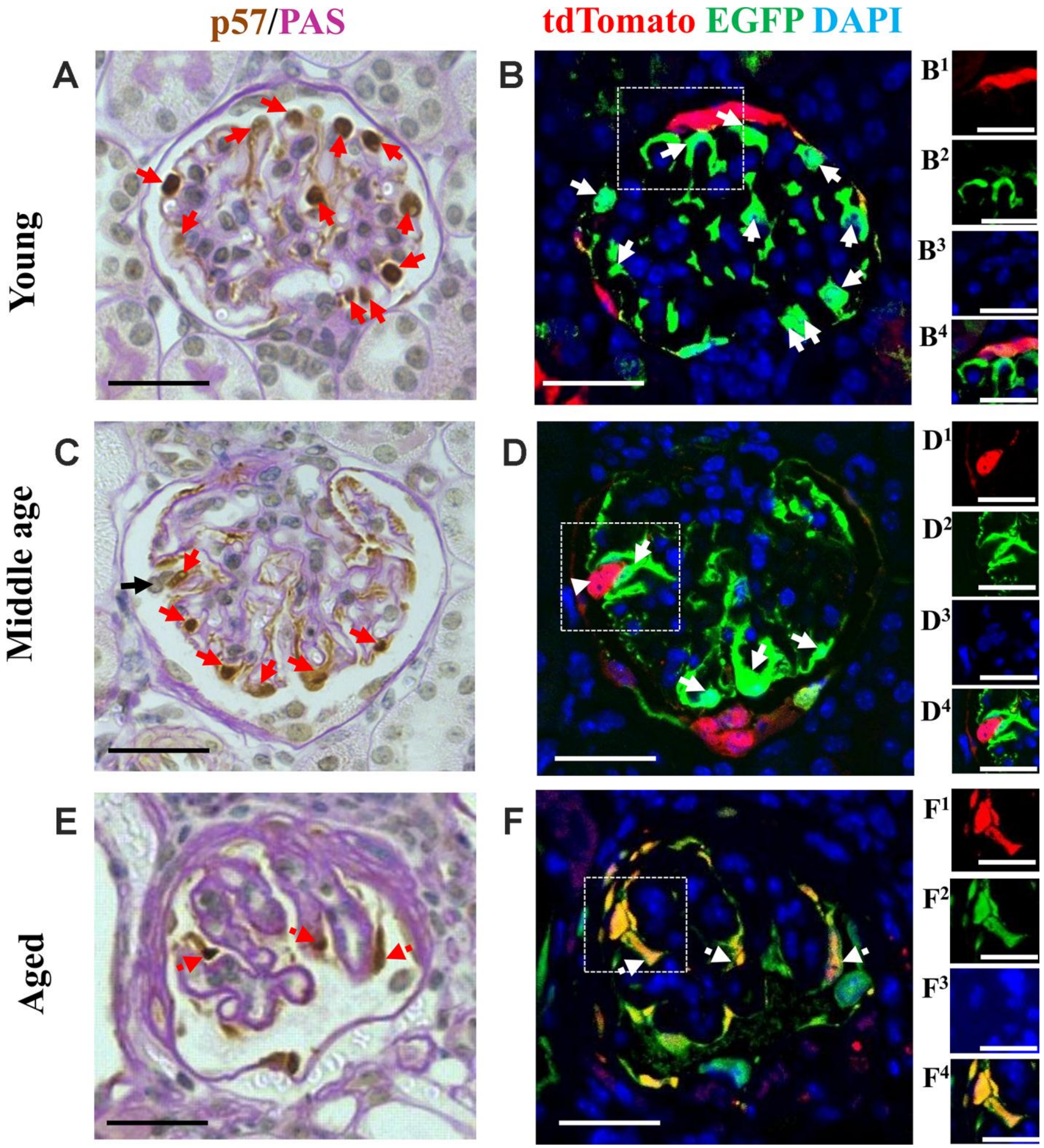

Supplementary Figure 2. Lower podocyte number was accompanied by a decrease in the podocyte specific EGFP reporter, and increased migration and differentiation of tdTomato+PECs to a podocyte fate in aged kidneys. (**A**–**F**) Paraffin embedded kidney sections from young, middle age and aged groups of mice were stained for podocyte marker p57 with PAS counterstain (**A**, **C**, **E**) and imaged. Subsequently, the same sections were stained for RFP (tdTomato reporter) and EGFP (podocyte reporter) (**B**, **D**, **F**). Small inserts show individual channels (1-red, 2-greeen, 3-far red) and merge of the area outlined by the white dashed boxes. (**A**, **B**) Young mice showed typical distribution of p57 stained nuclei (marked with red arrows) (**A**) Immunofluorescent image of the same glomerulus showed that majority of p57+ cells co-express EGFP reporter (**B**, **B**^{2}, **B**^{4}) (marked with white arrows), RFP-labeled PECs localized along BC (**B**, **B**^{1}, **B**^{4}), nuclei labeled with DAPI (**B**, **B**^{3}, **B**^{4}). (**C**, **D**) Middle age mice showed that decrease in P57+ podocytes (**C**) (marked with red arrows) accompanied a decrease in EGFP (**D**, **D**^{2}, **D**^{4}) (marked with white arrows). This was accompanied by migration of RFP labeled PECs (**D**, **D**^{1}, **D**^{4}) to the glomerular tuft (marked with arrow head) and segmental decrease in DAPI staining (**D**, **D**^{3}, **D**^{4}). (**E**, **F**) Aged mice showed that decrease in p57+ cells (**E**) (marked with dashed red arrows) accompanied a decrease in EGFP. This was also accompanied by migration of RFP labeled PECs (**F**, **F**^{2}, **F**^{4}) (marked with white dashed arrows) to the glomerular tuft and expression of EGFP, suggesting that migrated tdTomato$^+$PECs differentiate towards podocyte phenotype in aged kidneys. Scale bars represent 25µm or 5µm (insets).

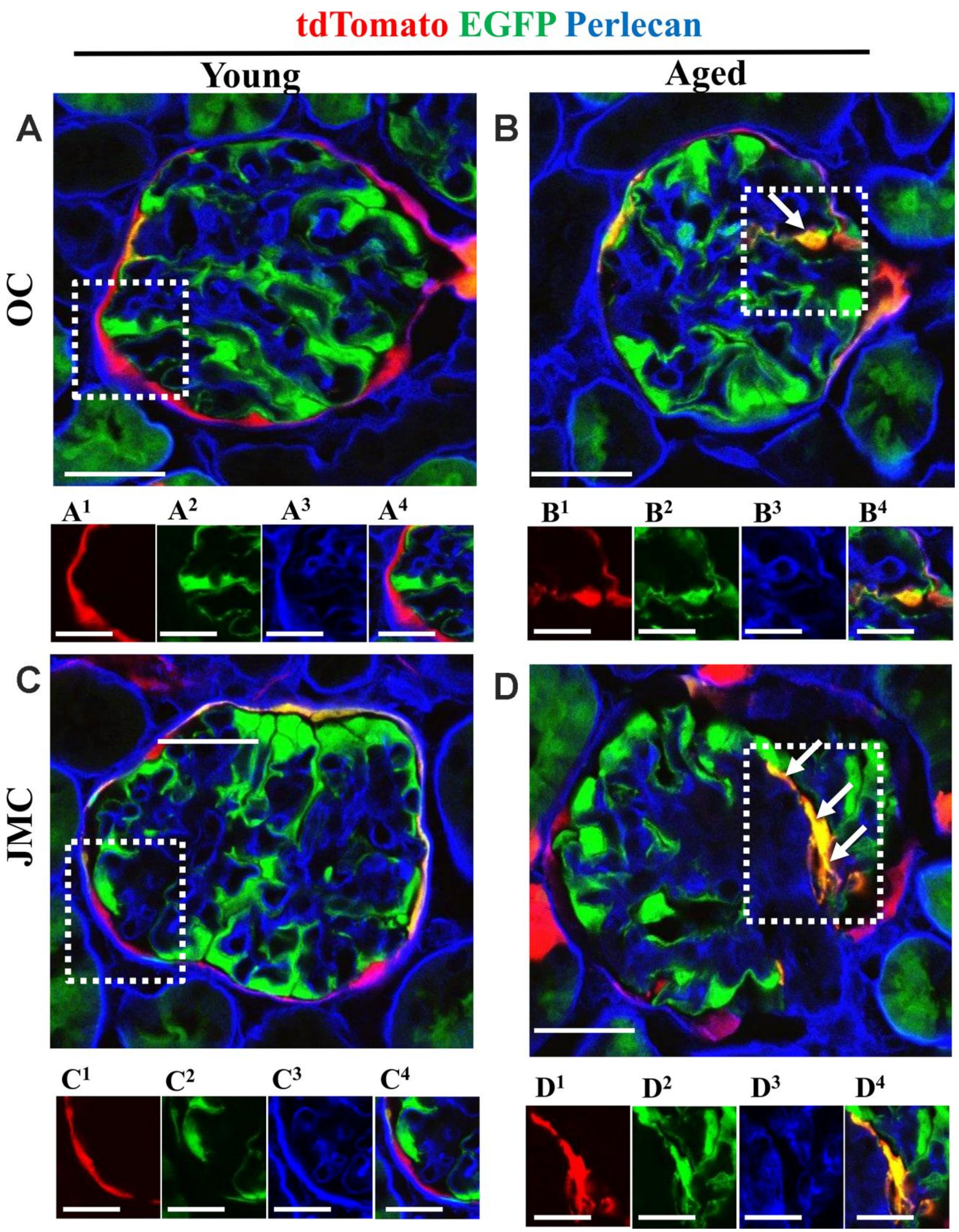

Supplementary Figure 3. Newly generated podocytes from PEC origin (tdTomato^{+}EGFP^{+}) are not mesangial cells. (**A**–**D**) Frozen kidney samples from young and aged groups of mice are stained for mesangial cell marker Perlecan (blue). Permanently labeled PECs (tdTomato reporter) and EGFP reporter are detected without the use of an antibody. Small inserts show individual channels (1-red, 2-greeen, 3-far red) and merge of the area outlined by the white dashed boxes. (**A**) Young mice (OC) showed that Perlecan (**A^{3}**) is expressed in mesangial cells, and is not detected in tdTomato^{+}PECs (**A^{1}**) or podocytes (EGFP^{+} cells) (**A^{2}**). (**B**) Aged mice (OC) showed that Perlecan (**B^{3}**) is not detected in migrated and differentiated yellow tdTomato^{+}EGFP^{+} cells (**B^{1}**, **B^{2}**) in the glomerular tuft (marked with solid arrow). (**C**) Young mice (JMC) showed that Perlecan staining (**C^{3}**) is limited to mesangial cells with no overlap with tdTomato^{+}PECs (**C^{1}**), or podocytes (EGFP^{+} cells) (**C^{2}**). (**D**) Aged mice (JMC) showed that the intensity of Perlecan staining (**D^{3}**) decreased in the glomerular tuft, but with no expression in yellow tdTomato^{+}EGFP^{+} cells (marked with solid arrows) (**D^{1}**, **D^{2}**). Scale bars represent 25µm or 5µm (insets).

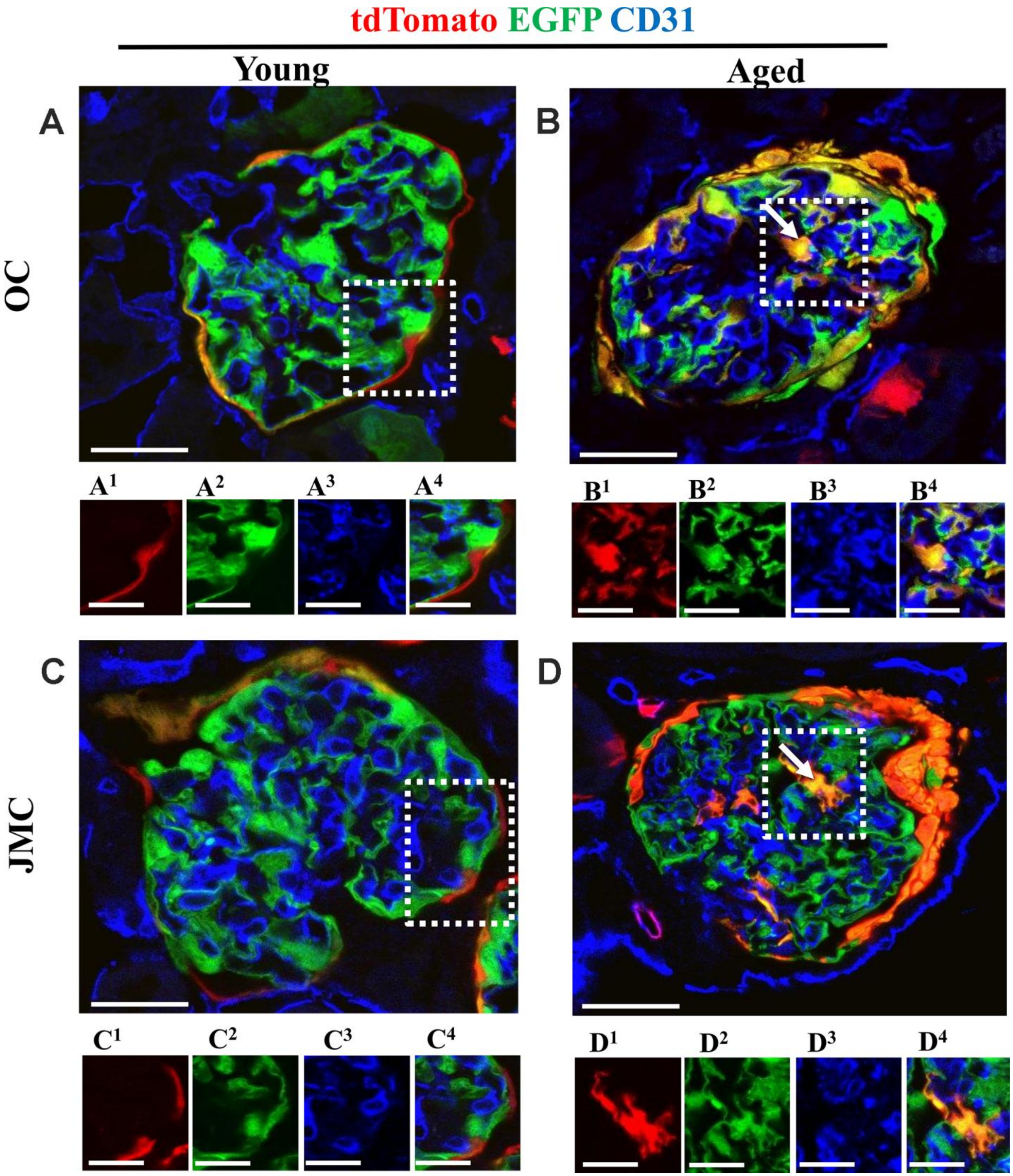

Supplementary Figure 4. Newly generated podocytes from PEC origin (tdTomato+EGFP+) are not endothelial cells. (**A**–**D**) Representative confocal images of tdTomato (red), EGFP+ (green), and CD31 (blue) in young and aged mice. Small inserts show individual channels (1-red, 2-greeen, 3-far red) and merge of the area outlined by the white dashed boxes. (**A**) Young mice (OC) showed that CD31 (**A**[3]) staining is limited to endothelial cells, and is not detected in tdTomato+PECs (**A**[1]) or podocytes (EGFP+ cells) (**A**[2]). (**B**) Aged mice (OC) showed that CD31 (**B**[3]) is not detected in migrated yellow tdTomato+EGFP+ cells (**B**[1], **B**[2]) in the glomerular tuft (marked with white arrow). (**C**) Young mice (JMC) showed that CD31 staining (**C**[3]) is detected in a typical endothelial distribution and does not show overlap with tdTomato+PECs (**C**[1]), or podocytes (EGFP+ cells) (**C**[2]). (**D**) Aged mice (JMC) showed that migrated yellow tdTomato+EGFP+ cells (**D1**, **D**[2]) do not co-express the endothelial marker CD31 (**D**[3]) in the glomerular tuft (marked with white arrow). Scale bars represent 25µm or 5µm (insets).

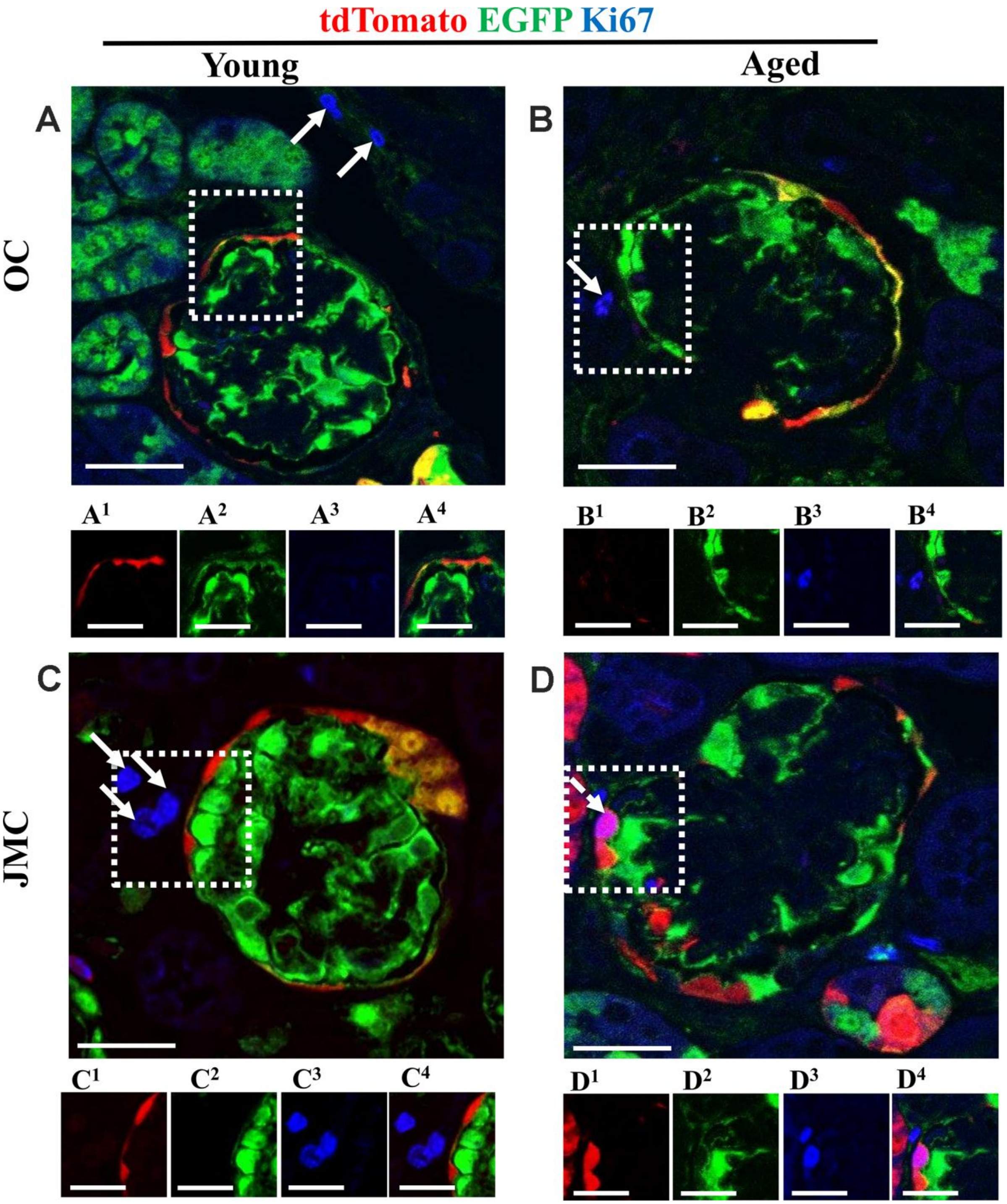

Supplementary Figure 5. Migrated tdTomato+PECs do not proliferate in the glomerular tuft of aged mice Representative images of tdTomato (red, Pec reporter), EGFP (green, podocyte reporter), and Ki67 (far-red, proliferation marker). Small inserts show individual channels (1-red, 2-greeen, 3-far red) and merge of the area outlined by the white dashed boxes. (**A**) Young mice (OC) showed that occasional Ki67+ cells (**A**^{3}) were observed in the tubular compartment (labeled with solid arrows). tdTomato+PECs (**A**^{1}) and EGFP+ podocytes (**A**^{2}) and do not co-express Ki67 in the glomerular tuft. (**B**) Aged mice (OC) showed that double positive tdTomato+EGFP+ cells (**B**^{1}, **B**^{2}) do not overlap with Ki67 (B3) in the glomerulus. Occasional tubular cells stained with Ki67+ (marked with white arrow). (**C**) Young mice (JMC) showed that Ki67 staining (**C**^{3}) was detected in tubular cells (marked with white arrows). EGFP+ podocytes (**A**^{2}) and tdTomato+PECs (**C**^{1}) do not co-express Ki67 in the glomerular tuft. (**D**) Aged mice (JMC) showed that Ki67 (**D**^{3}) was occasionally expressed in tdTomato+PECs (marked with dashed arrow) along BC, tdTomato+PECs that migrated to the glomerular tuft (**D**^{1}) and EGFP+ podocytes (**D2**) did not overlap with Ki67. Scale bars represent 25µm or 5µm (insets).
